# Isoniazid Resistance in *Mycobacterium tuberculosis* Is a Heterogeneous Phenotype Composed of Overlapping MIC Distributions with Different Underlying Resistance Mechanisms

**DOI:** 10.1128/AAC.00092-19

**Published:** 2019-06-24

**Authors:** Arash Ghodousi, Elisa Tagliani, Eranga Karunaratne, Stefan Niemann, Jennifer Perera, Claudio U. Köser, Daniela Maria Cirillo

**Affiliations:** a Emerging Bacterial Pathogens Unit, Division of Immunology, Transplantation and Infectious Diseases, IRCCS San Raffaele Scientific Institute, Milan, Italy; b Faculty of Medicine, University of Colombo, Colombo, Sri Lanka; c Molecular and Experimental Mycobacteriology, Research Center Borstel, Borstel, Germany; d German Center for Infection Research, Partner site Hamburg-Lübeck-Borstel-Riems, Germany; e Department of Genetics, University of Cambridge, Cambridge, United Kingdom

**Keywords:** MGIT 960, MIC, *Mycobacterium tuberculosis*, isoniazid resistance, whole-genome sequencing

## Abstract

MIC testing using the Bactec mycobacteria growth indicator tube system 960 of 70 phylogenetically diverse, isoniazid-resistant clinical strains of Mycobacterium tuberculosis revealed a complex pattern of overlapping MIC distributions. Whole-genome sequencing explained most of the levels of resistance observed. The MIC distribution of strains with only *inhA* promoter mutations was split by the current concentration endorsed by the Clinical and Laboratory Standards Institute to detect low-level resistance to isoniazid and is, consequently, likely not optimally set.

## Abstract (precis)

MIC testing using the Bactec mycobacteria growth indicator tube system 960 of 70 phylogenetically diverse, isoniazid-resistant clinical strains of Mycobacterium tuberculosis revealed a complex pattern of overlapping MIC distributions. Whole-genome sequencing explained most of the levels of resistance observed.

## TEXT

In light of the continued selection and spread of drug-resistant tuberculosis, coupled with the dearth of novel antibiotics, the question of whether low-level resistance can be overcome by increasing the dose of a drug has become increasingly urgent (1). In 2018, the World Health Organization (WHO) formally endorsed this possibility for moxifloxacin, whereby a dose of 800 mg/day can be used to treat low-level resistance to this fluoroquinolone, although the corresponding clinical breakpoint has not been recognized by the Clinical and Laboratory Standards Institute (CLSI) (2–5). Conversely, for at least 15 years, the position of CLSI has been to stratify resistance to the core drug isoniazid (INH) into low- and high-level resistance by testing two concentrations of this drug, whereas WHO has not endorsed this concept to date (6–8). Specifically, the CLSI recommendation is to include the following statement in the antimicrobial susceptibility testing (AST) reports of strains that are only low-level resistant (i.e., are resistant to INH at the critical concentration [CC] of 0.1 μg/ml but not the higher clinical breakpoint of 0.4 μg/ml): “A specialist in the treatment of [multidrug-resistant tuberculosis] should be consulted concerning the appropriate therapeutic regimen and dosages” (3). However, WHO is in the process of reviewing its recommendation for INH and, in its most recent manual for AST, has begun to stratify INH resistance on the genotypic level but has not yet set corresponding clinical breakpoints to align the phenotype (7). We, therefore, set out to compare the phenotypic definitions of low- and high-level resistance of CLSI with the genotypic stratification proposed by WHO.

To this end, we used the BC Bactec mycobacteria growth indicator tube (MGIT) 960 system to conduct comprehensive MIC testing of a select set of phylogenetically diverse strains (70 INH-resistant and 5 INH-susceptible isolates), along with Mycobacterium tuberculosis H37Rv ATCC 27294 as the control strain. Four serial 2-fold dilutions were prepared from an INH stock solution to provide a final test range of 0.016 to 0.25 μg/ml for susceptible strains and H37Rv, 0.25 to 4 μg/ml for *inhA* promoter mutant isolates with or without a concurrent *inhA* coding mutation, 1 to 16 μg/ml for S315T/N mutant isolates, and 4 to 64 μg/ml for isolates with double mutations in *katG* and the *inhA* promoter. Whole-genome sequencing (WGS) was carried out with the Nextera-XT DNA kit to prepare paired-end libraries of 150-bp read lengths for Illumina sequencing. Data analysis and single-nucleotide polymorphism calling were performed using the MTBseq pipeline (9) (for additional details, see Supplementary methods in the supplemental material).

**FIG 1:**
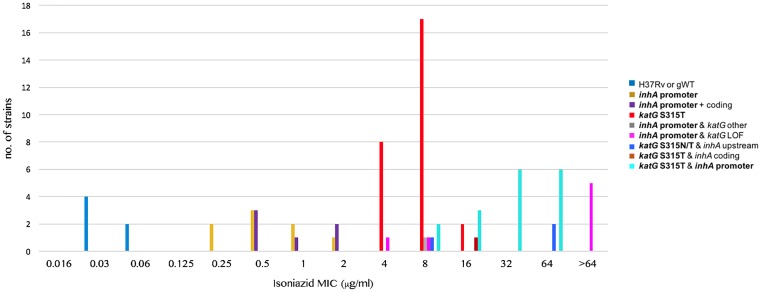
Isoniazid MIC results stratified by known or likely resistance mutations in the coding region of *katG* or *inhA* or mutations that result in the overexpression of *inhA*. All of the latter mutations are upstream of *inhA*, but “promoter” is used to highlight mutations in the −16 to −8 region upstream of the transcriptional start site of the *fabG1-inhA* operon, which can be detected by the WHO-endorsed Hain GenoType MTBDR*plus* version 2 and Nipro NTM+MDRTB version 2 assays (all mutations interrogated by these assays are shown in bold in the key of the plot [25]). gWT, genotypically wild-type strain (i.e., strain without known resistance mutations); LOF, loss-of-function mutation (i.e., insertion, deletion, or nonsense mutation).

The six susceptible controls had INH MICs of 0.03 to 0.06 μg/ml (Fig. 1 and Table S2 in the supplemental material). In contrast, resistant strains displayed a series of overlapping MIC distributions. Strains that had only mutations that are interrogated by the WHO-endorsed genotypic AST assays (i.e., the Hain GenoType MTBDR*plus* version 2 and Nipro NTM+MDRTB version 2) resulted in three MIC distributions (10), i.e., strains that had only an *inhA* promoter change or a mutation at codon 315 of *katG* had nonoverlapping MIC distributions of 0.25 to 2 and 4 to 16 μg/ml, respectively, and strains with both mutations displayed MICs of 8 to 64 μg/ml. The variation in these distributions was likely largely due to the normal variation in MIC testing (i.e., even in the same laboratory, a variation of plus or minus one dilution is inevitable, which is further exacerbated by the variation in testing between laboratories).

The precise level of resistance cannot be predicted by using the Hain and Nipro assays alone because mutations that are not interrogated by these assays can increase the MICs. To some extent, this can be overcome by using WGS data, provided that known mutations with predictable effects are identified (i.e., the level of resistance could not be fully explained even with WGS). For example, some but not all strains with loss-of-function mutations in *katG* had MICs of >64 μg/ml (Fig. 1). Moreover, a C deletion 34 nucleotides upstream of the main transcriptional start site of *inhA* likely accounted for the unusually high MIC of 64 μg/ml for the *katG* S315N mutant (11). In contrast, another mutation upstream of *inhA* at codon 203 of *fabG1*, which is known to result in the overexpression of *inhA* by creating an alternative promoter, did not appear to increase the MIC above the level explained by the *katG* S315T mutation in the strain in question (i.e., 8 μg/ml) (12). Similarly, there was an almost complete overlap between the MIC distributions of strains that harbored only *inhA* promoter mutations and those that had additional *inhA* coding mutations at codon 21, 94, or 194 (i.e., 0.25 to 2 μg/ml versus 0.5 to 2 μg/ml).

This study was conducted in a single center, so we could not quantify the effect of laboratory-to-laboratory variation. Data from additional laboratories are needed to define robust quality-control ranges/targets to evaluate whether the current CC of 0.1 μg/ml corresponds to the epidemiological cutoff and to define the lower and upper ends of the various resistance mechanisms more accurately (3, 7, 13). Moreover, breakpoints cannot be set based on MIC data alone (5, 14, 15). Nevertheless, the MIC distributions in this study have implications for defining low-level resistance. The current clinical breakpoint of CLSI to define low-level resistance (i.e., 0.4 μg/ml, which corresponds to 0.5 μg/ml using our dilution series) does not correspond to the upper end of the MIC distribution of *inhA* promoter mutants. Instead, the upper end of the MIC distribution of a mechanism has to be considered when assessing whether it is treatable with either the standard or an elevated dose of INH (16–18). For strains with only *inhA* promoter mutations, this target concentration would be 1 or 2 μg/ml (i.e., at least 10 times higher than the current CC) (3, 7). Should pharmacokinetic/pharmacodynamic, drug penetration, and clinical outcome data confirm that this target is achievable, 1 or 2 μg/ml may be adopted instead of 0.4 μg/ml (14, 15). This would avoid splitting the MIC distribution of *inhA* promoter mutants and would, consequently, reduce or eliminate their misclassification as high-level resistant because of the technical variation in AST, as is the case with the current clinical breakpoint of CLSI.

One argument against setting a clinical breakpoint at 1 or 2 μg/ml might be that it would result in the misclassification of strains with both *inhA* promoter and coding mutations as low-level resistant, as stressed in a previous consensus statement (7, 19). However, two aspects should be borne in mind. First, only 3% (95% confidence interval, 1% to 6%) of strains with *inhA* promoter mutations in the −16 to −8 region that do not have *katG* mutations have additional *inhA* coding mutations based on recent WHO population-level surveillance data from seven countries (20). Therefore, in most settings, misclassifications of double mutants would be rare compared with the increased ability to detect *inhA* promoter mutants with a higher clinical breakpoint. Second, the effect of these coding mutations on the INH MIC and thus clinical outcome is likely modest at worst, but more MIC data are needed for the mutations at different *inhA* codons (21–24). Nonetheless, it might be advisable for countries that conduct routine WGS to err on the side of caution by classifying these double mutants as high-level resistant until clinical data to the contrary are available, although, in practice, it would be challenging to conduct a sufficiently powered study to address this question because these mutations are so rare.

## Supplementary Material

Supplemental file 1

Supplemental file 2
