# Supplementary material for: Isoniazid Resistance in Mycobacterium tuberculosis Is a Heterogeneous Phenotype Composed of Overlapping MIC Distributions with Different Underlying Resistance Mechanisms
Source: Antimicrob Agents Chemother. 2019 Jun 24;63(7):e00092-19. doi: 10.1128/AAC.00092-19 (PMC6591585; doi:10.1128/AAC.00092-19)
Supplement: Supplemental file 1 [file AAC.00092-19-s0001.pdf]

Supplementary methods

MIC testing for isoniazid was performed with the BACTEC 960 MGIT as recommended by the manufacturer at the San Raffaele Scientific Institute, Milan (Italy). The details of all strains included in this study as well as the precise concentrations tested for each can be found in Table S1. Control tubes without antibiotic were inoculated with 1:100 diluted bacterial suspensions. The MIC was defined as the lowest antibiotic concentration that completely inhibited the growth when the 1:100 controls reached a GU (gas unit) of 400. Genomic DNA was extracted by the cetyl-trimethyl ammonium bromide (CTAB) method from Löwenstein-Jensen cultures and was used for whole-genome sequencing using a modified Illumina NexteraXT protocol and the MiSeq or NextSeq sequencers. The raw data (fastq files) was submitted to the European Nucleotide Archive (Table S1). Data analysis, SNPs calling and lineage/sub-lineage classification were performed using the MTBseq pipeline (1), BWA (2), SAMtools (3), PICARD-tools (https://broadinstitute.github.io/picard/), and the Genome Analysis Toolkit (4). The genome of strain H37Rv (NC-000962.3) was used as a reference. To detect possible resistant subpopulations, the MTBseq was launched with parameters adjusted. We focused our analysis on the five genes that are known to provide the greatest sensitivity for isoniazid resistance (Table S2), from which known polymorphisms that do not correlate with resistance were excluded (i.e. *katG* 2154724 Arg463Leu and *ahpC* promoter 2726105 G-88A (5). The genotype for each genome was assigned using the Coll nomenclature (6).

Table S1. Genomic coordinates of gene regions analyzed in this study.

Gene	Locus	Genomic coordinates
katG coding	*Rv1908c*	2153889-2156111
furA-katG intergenic region		2156112-2156148
furA promoter+coding	*Rv1909c*	2156149-2156692
fabG1 promoter+coding	*Rv1483*	1673340-1674183
fabG-inhA intergenic region		1674184-1674201
inhA coding	*Rv1484*	1674202-1675011
ahpC promoter+coding	*Rv2428*	2726088-2726780

References

1. Kohl TA, Utpatel C, Schleusener V, De Filippo MR, Beckert P, Cirillo DM, Niemann S. 2018. MTBseq: a comprehensive pipeline for whole genome sequence analysis of *Mycobacterium tuberculosis* complex isolates. PeerJ 6:e5895.
2. Li H, Durbin R. 2009. Fast and accurate short read alignment with Burrows-Wheeler transform. Bioinformatics 25:1754-60.
3. Li H, Handsaker B, Wysoker A, Fennell T, Ruan J, Homer N, Marth G, Abecasis G, Durbin R. 2009. The Sequence Alignment/Map format and SAMtools. Bioinformatics 25:2078-9.
4. McKenna A, Hanna M, Banks E, Sivachenko A, Cibulskis K, Kernytsky A, Garimella K, Altshuler D, Gabriel S, Daly M, DePristo MA. 2010. The Genome Analysis Toolkit: a MapReduce framework for analyzing next-generation DNA sequencing data. Genome Res 20:1297-303.
5. Feuerriegel S, Köser CU, Niemann S. 2014. Phylogenetic polymorphisms in antibiotic resistance genes of the *Mycobacterium tuberculosis* complex. J Antimicrob Chemother 69:1205-10.
6. Coll F, McNerney R, Guerra-Assuncao JA, Glynn JR, Perdigão J, Viveiros M, Portugal I, Pain A, Martin N, Clark TG. 2014. A robust SNP barcode for typing *Mycobacterium tuberculosis* complex strains. Nat Commun 5:4812.
